# The completeness of national hip and knee replacement registers

**DOI:** 10.2340/17453674.2024.42303

**Published:** 2024-11-18

**Authors:** Jonathan M R FRENCH, Kevin DEERE, Michael R WHITEHOUSE, Derek J PEGG, Enrico CIMINELLO, Riccardo VALENTINI, Marina TORRE, Keijo MÄKELÄ, Anne LÜBBEKE, Eric R BOHM, Anne Marie FENSTAD, Ove FURNES, Geir HALLAN, Jinny WILLIS, Søren OVERGAARD, Ola ROLFSON, Adrian SAYERS

**Affiliations:** 1 Musculoskeletal Research Unit, University of Bristol Medical School, Bristol, UK; 2 National Institute for Health Research, Bristol Biomedical Research Centre, University Hospitals Bristol and Weston NHS Foundation Trust and University of Bristol, UK; 3 Mid Cheshire Hospitals Foundation Trust, Leighton Hospital, Crewe, UK; 4 Italian National Institute of Health, Rome, Italy; 5 Turku University Hospital, and University of Turku, Turku, Finland; 6 Geneva University Hospitals and Geneva University, Switzerland; 7 Nuffield Department of Orthopaedics, Rheumatology and Musculoskeletal Sciences, University of Oxford, Oxford, UK; 8 University of Manitoba, Winnipeg, Canada; 9 Norwegian Arthroplasty Register, Department of Orthopaedic Surgery, Haukeland University Hospital, Bergen, Norway; 10 Department of Clinical Medicine, University of Bergen, Bergen, Norway; 11 New Zealand Joint Registry, Christchurch Hospital, New Zealand; 12 Department of Orthopaedic Surgery and Traumatology, Copenhagen University Hospital, Bispebjerg, Denmark; 13 University of Copenhagen, Department of Clinical Medicine, Faculty of Health and Medical Sciences, Denmark; 14 Institute of Clinical Sciences, University of Gothenburg, Gothenburg, Sweden

## Abstract

**Background and purpose:**

National joint replacement registries were developed for prospective monitoring of outcomes and post-market surveillance of implants. Increasingly registry data informs practice. However, analysis of a registry can only be as good as the data it captures on the population of interest. We aimed to analyze completeness of reporting of hip and knee replacement procedures for all national registries worldwide.

**Methods:**

We analyzed annual reports and data provided following written requests to all active national hip and knee replacement registries. Coverage was defined as the proportion of hospitals in the country that participate in the registry. Procedure completeness was defined as the proportion of procedures successfully captured by the registry.

**Results:**

14 national registries were included, spanning years 2004 to 2022. Coverage was complete in 10. Median procedure completeness for primary hip and knee replacement across all years was 96.5% (interquartile range [IQR] 94.0–97.7%). Median procedure completeness for revisions was 88.5% (IQR 81.0–92.5%). The terminology used and method of calculation of completeness estimates in the registries were variable.

**Conclusion:**

National hip and knee replacement registry data generally reflects excellent coverage (full in 10 of 14 registries) and completeness (primary procedures 96.5% and revisions 88.5%) over the last 2 decades.

National joint replacement registries are used to prospectively monitor the short- and long-term outcomes—in terms of risks (mainly revision) and benefits (mainly patient-reported outcomes)—following joint replacement surgery. They provide the main mechanism for pre- and post-market surveillance of prostheses and benchmarking of established joint replacements [1,2]. Large patient numbers provide the sensitivity for early detection of issues with the outcomes of specific implants or techniques that are not performing well [3]. Increasingly the results of registry studies shape orthopedic practice in the choice of surgical approach, specific implants, component size, materials, and method of prosthesis fixation, where clinical trials might be impractical [4]. As the scope and influence of registry data grows, so does the importance of understanding its quality.

A national joint replacement registry functions as a prospective cohort study where the exposure is the primary joint replacement procedure, and the population is all patients within a country who have had the joint replacement of interest. A comprehensive population sample is the key feature of registries; there can be no sampling error if the entire population is captured, nor outcomes missed. Therefore, knowledge of the coverage and completeness of all registries is essential to interpreting the data.

Coverage and completeness are terms that are often used interchangeably [5-7]. However, for clarity in the context of national datasets it is important to differentiate between the 2 [8,9].

Coverage describes the proportion of hospitals that participate in the registry compared with the total number of hospitals performing procedures in the country (Table 1) [9].

**Table 1:** Definitions in context of national implant registries

Term	Definition
$\text{Coverage}\;\text{=}\;\frac{\text{Number}\;\text{of}\;\text{participating}\;\text{hospitals}\;\text{in}\;\text{the}\;\text{country}\;\text{or}\;\text{region}}{\text{Number}\;\text{of}\;\text{hospitals}\;\text{performing}\;\text{procedures}\;\text{in}\;\text{the}\;\text{country}\;\text{or}\;\text{region}}$	
$\text{Procedure}\;\text{completeness}\;\text{=}\;\frac{\text{Number}\;\text{of}\;\text{procedures}\;\text{registered}}{\text{True}\;\text{number}\;\text{of}\;\text{procedures}\;\text{performed}}$	

Completeness describes the number of procedures successfully captured by a registry as a proportion of the true number of procedures that occurred in the population [10]. As the true number cannot be known, national joint registries typically derive completeness from comparison with a national inpatient database [9,11,12]. Completeness estimates derived from comparison between 2 likely incomplete datasets should account for records potentially missed by both, with comparison and pooling of procedures identified by each dataset, and the possibility of additional epidemiological techniques employed, such as capture–recapture analysis [13]. Currently there is no standardized method for calculating completeness between national joint registries; it is important to understand the different methods used as these can impact results.

We therefore aimed to collate the coverage and completeness of hip and knee replacement procedures in all national joint registries, examine trends over time, and report the methods used to calculate them. In addition, we aimed to assess whether the method of calculation could be improved by comparison with estimates using capture–recapture methods where possible.

## Methods

### Data sources and collection

This study is reported according to the PRISMA guidelines.

National joint replacement registries were initially identified from the member list of the International Society for Arthroplasty Registries (ISAR) [14]. Member registry websites were individually evaluated for affiliate links to other registries, indexed, and combined with internet searches to ensure a comprehensive list. All available annual reports were downloaded and evaluated, with regular rounds of data collection ending in January 2024. Registries without reports or data available on their websites were contacted in writing on at least 2 separate occasions.

Inclusion criteria were national registries with at least 5 years of data available, either publicly available or through direct written request, which included reference to coverage, completeness, or data quality. Exclusion criteria were non-national, i.e., institutional or private-group registries.

Data was collected from annual reports on hip and knee replacement procedures including type, primary or revision surgery, registry coverage, procedural completeness by year (2004 to 2022), method by which completeness was calculated, and total number of cases in the registry. Registry annual report data was extracted by 2 reviewers independently (AS, KW), followed by checking and inclusion of data on request by JF. Data was further verified by relevant co-authors for their respective registry. There were no disputes in the data collection process requiring a referee.

Where completeness data were incomplete, ambiguous, or not presented in an annualized form, a written request was made to the registry. This was necessary for the New Zealand Joint Registry (NZJR), Dutch Joint Registry (LROI), Canadian Joint Replacement Registry (CJRR), Swiss National Hip & Knee Joint Registry (SIRIS), and American Joint Replacement Registry (AJRR), which all provided data directly bar the AJRR, which responded that it does not currently collect data on coverage or procedural completeness (Table 2).

**Table 2:** List of registries included in the study, acronyms, and source of data

Acronym	Registry	Data extracted from	
		Annual reports	Written request
AJRR	American Joint Replacement Registry (USA)		√
AOANJRR	Australian Orthopaedic Association National		
	Joint Replacement Registry	√	
CJRR	Canadian Joint Replacement Registry	√	√
DHR	Danish Hip Arthroplasty Register	√	
DKR	Danish Knee Arthroplasty Register	√	
EPRD	Endoprothesenregister Deutschland (Germany)	√	
FAR	Finnish Arthroplasty Register	√	
LROI	Landelijke Registratie Orthopedische Interventies (Netherlands)	√	√
NAR	Norwegian Arthroplasty Register	√	
NJR	National Joint Registry (England and Wales)	√	
NZJR	New Zealand Joint Registry		√
RIAP	Registro Italiano ArtroProtesi (Italy)	√	√
SAR	Swedish Arthroplasty Register	√	
SIRIS	Schweizerisches Implantat-Register/Registre Suisse des Implants (Switzerland)	√	√

Where possible, completeness analyses were grouped by procedure and whether primary or revision. This was decided a priori as completeness was expected to be lower for revisions due to its potential emergency nature and reliance on successful procedural linkage.

### Statistics

Descriptive statistics and generation of graphs were performed using Stata (version 18, StataCorp LLC, College Station, TX, USA). Summary statistics are presented as median and interquartile ranges (IQR). Capture–recapture analysis was performed using the Lincoln–Peterson method for 2 lists (see Appendix) [13]. The method uses 2 incomplete samples from the same population to estimate the true total population size, performed here to provide a comparison to test the validity of current national joint registry completeness calculations.

### Ethics, registration, data sharing, funding, and disclosures

Ethical review board approval for this research was deemed not necessary as publicly available data was used. The study was not registered. All data will be shared upon request. This study was supported by the NIHR Biomedical Research Centre at University Hospitals Bristol and Weston NHS Foundation Trust, the University of Bristol, Orthopaedic Research UK (ORUK), and the British Hip Society (BHS). The views expressed are solely of the authors and not necessarily those of the NIHR, the Department of Health and Social Care, ORUK, or BHS. Several authors have leadership positions in joint registries and/or the International Society of Arthroplasty Registries (MRW, DJP, MT, KM, AL, ERB, AMF, OF, GH, OR, AS, SO). MRW is the chief investigator for the lot 2 contract for the National Joint Registry (NJR): Statistical Support, Analysis and Associated Services. KD and AS are part of the same contract. JMF is a clinical research fellow funded by Orthopaedic Research UK and the British Hip Society. The views expressed herein are solely those of the authors. Complete disclosure of interest forms according to ICMJE are available on the article page, doi: 10.2340/17453674.2024.42303

## Results

**Figure 1:**
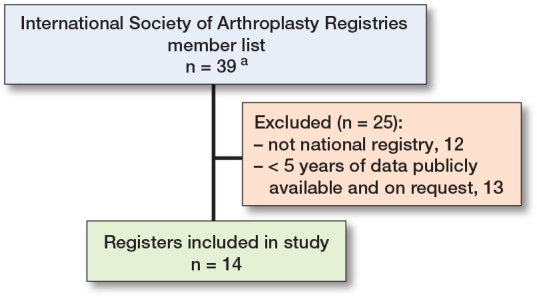
Flowchart showing study registry inclusion. ^a^ 14 were full members and 25 were affiliate members.

14 national joint replacement registries were identified for inclusion in the study (Figure 1, Table 2) [11,12,15-26], containing over 13 million procedures. Of these registries, 10 had full coverage: the Australian Orthopaedic Association National Joint Replacement Registry (AOANJRR), Danish Hip Arthroplasty Register (DHR), Danish Knee Arthroplasty Register (DKR), Finnish Arthroplasty Register (FAR), LROI, NJR, NZJR, Norwegian Arthroplasty Register (NAR), SIRIS, and Swedish Arthroplasty Register (SAR). Among these, all bar AOANJRR and SAR had mandatory submission. These registries had the highest completeness for hip and knee procedures. In Canada (CJRR) and Germany (EPRD), registry participation is available to all centers nationally on a partial or fully voluntary basis [16,27]. In Italy the completeness of RIAP is about 35%, with historic fluctuations where key regions did not submit data [12]. Coverage and completeness data is not yet collected by the AJRR (USA) [25]. 3 registries used the terms coverage and completeness interchangeably [17,23,27]. Other terms such as capture-rate and compliance were also used to describe completeness [15,21].

**Figure 2:**
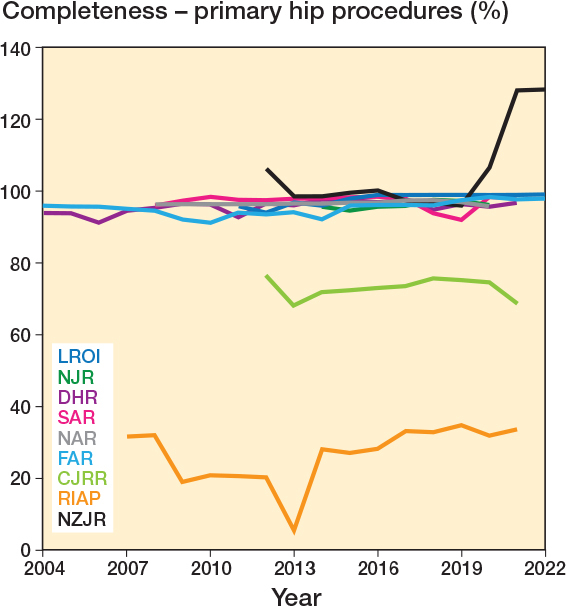
Yearly completeness for primary hip procedures by registry. CJRR data includes both primary hip and knee procedures. See Results and Discussion for explanation of completeness values of more than 100%.

**Figure 3:**
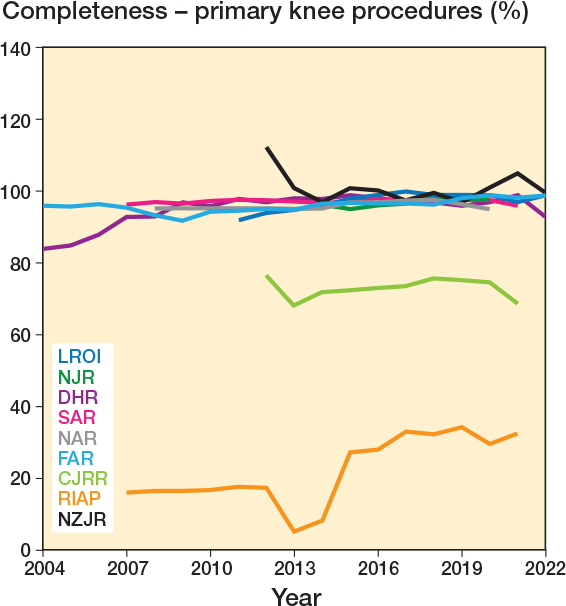
Yearly completeness for primary knee procedures by registry. CJRR data includes both primary hip and knee procedures. Also see Legend to Figure 2.

Completeness data was recorded by 13 registries to varying levels of detail between years 2003 and 2021. 10 registries provided completeness data specifically for primary procedures, with median completeness of 96.5% (IQR 94.0–97.7, 230 observations). Primary completeness data comprised 8 registries that reported specifically on primary hip replacement, median 96.5% (IQR 94.2–97.8; Figure 2), and 8 registries that reported specifically on primary knee replacement, median completeness 96.7% (IQR 94.9–98.0; Figure 3).

**Figure 4:**
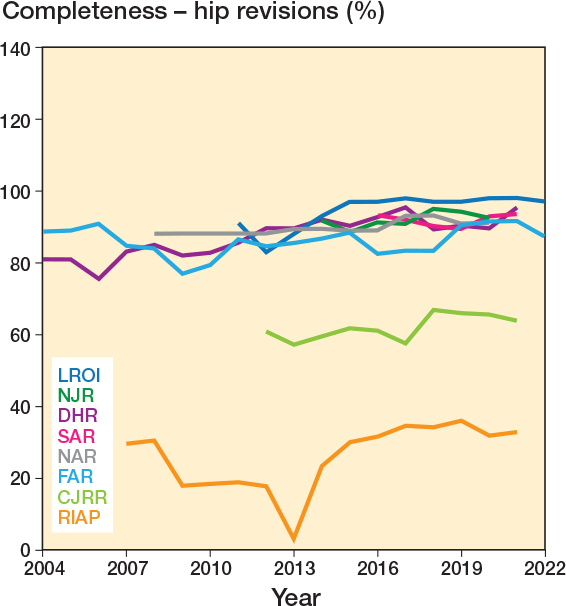
Yearly completeness for hip revisions by registry. CJRR data includes both revision hip and knee procedures.

**Figure 5:**
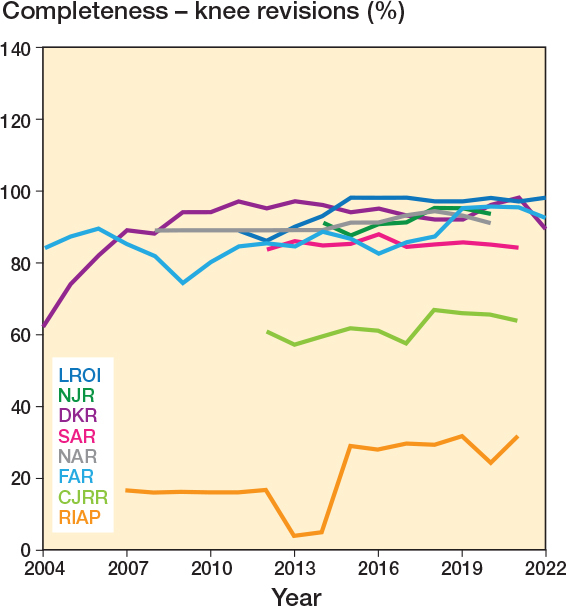
Yearly completeness for revision knee procedures, by registry. CJRR data includes both revision hip and knee procedures.

Completeness was reported for revisions by 9 registries, median 88.5% (IQR 81.0–92.5, 195 observations). These are displayed in Figures 4 and 5, where a general upward trend can be seen following registry inception. Specific data on hip-revision completeness was provided by 7 registries, with a median of 89% (IQR 82.8–91.9; Figure 4). Specific knee-revision completeness was provided by 7 registries, median 88.9% (IQR 83.8–94.0; Figure 5).

**Figure 6:**
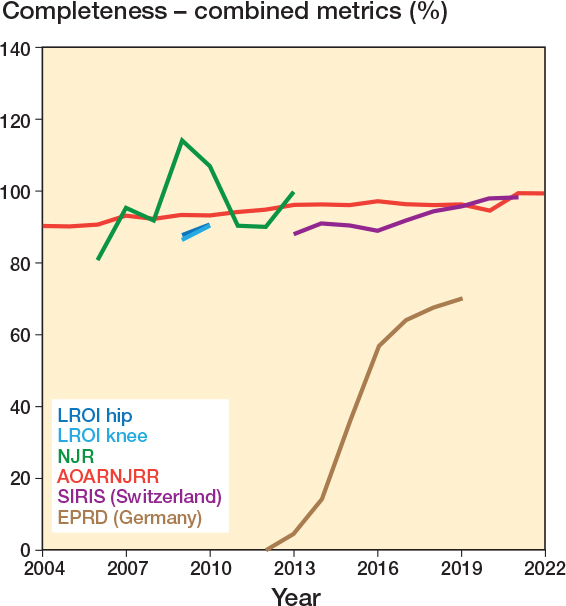
Yearly completeness for registries that reported primary and revision procedures as a combined metric. Also see Legend to Figure 2.

At various timepoints a total of 5 registries grouped together primary and revision procedures, and hip and knee replacements, when reporting completeness. Median completeness for these was 93.0% (IQR 89.9–96.0%, 57 observations). This is displayed in Figure 6, where similarly an upward trend over time can be observed.

The method of completeness calculation is summarized in Table 3. 6 registries used matched datasets by procedure, with the union of the 2 (or more) datasets forming the reference population and therefore denominator for the completeness calculation. 7 registries simply divided the number of registry procedures by the number in the reference dataset, typically a national hospital patient database. This latter method assumes the reference dataset to be complete and correct and resulted in instances of purported completeness values of more than 100% (Figures 2, 3, 6), which were reported by 2 registries, the NJR and NZJR. The NZJR calculates completeness using the number of procedures coded in the national hospital patient database as the denominator. Prior to 2014 the NJR calculated completeness using the number of implants purchased by all participating hospitals that year as the denominator, resulting in values for the calculated completeness of over 100% when implants had been stockpiled.

**Table 3:** Registry coverage and method of completeness calculation

Registry	Coverage and participation	Registry procedures and completeness calculation method	Matched data specified
AJRR	Unknown; voluntary	Not currently calculated	
AOANJRR	Nationwide; voluntary	Union of government database + registry procedures with 3-step verification process	√
CJRR	Partial; partially mandatory **^a^**	Hospital patient database (DAD/HMDB and NACRS) + registry procedures	
DHR, DKR	Nationwide; mandatory	Union of hospital patient database (LPR) + registry procedures	√
EPRD	Partial; voluntary	Procedures estimated from national quality assurance reports (AQUA and IQTIG)	
FAR	Nationwide; mandatory	Hospital patient database (HDR) procedures	
LROI	Nationwide; mandatory	Hospital patient database (HIS) procedures	
NAR	Nationwide; mandatory **^b^**	Union of hospital patient episode database (NPR) + registry procedures	√
NJR	Nationwide; mandatory	Union of hospital patient database (HES/PAS) + registry procedures **^d^**	√
NZJR	Nationwide; mandatory	Registry procedures/hospital patient database (NMDS) procedures	
RIAP	Partial; partially mandatory **^c^**	Procedures registered in the national hospital discharge database (HDD)	
SAR	Nationwide; voluntary	Union of hospital patient database (NPR) + registry procedures	√
SIRIS	Nationwide; mandatory	Public health figures (BAG)	

a For 3 provinces and 2 regions, voluntary elsewhere.

b Provided patients consent.

c For 5 regions, voluntary elsewhere.

d Previously were registry procedures = implants purchased, which gave values > 100% when implants were stockpiled, as seen in Figure 3.

1 registry (NAR) published sufficient data in its annual report to allow a comparative completeness estimate using capture–recapture methodology [22]. For primary hip procedures in 2019–2020 (2023 report) completeness was reported as 97.0%; the estimate using capture–recapture was 96.8%. For hip revisions the completeness was reported as 90.8% from a total population of 3,178 procedures; capture–recapture produced an adjusted estimate of 87.5% (see Appendix 1 for full calculations).

## Discussion

We aimed to analyze completeness of reporting of hip and knee replacement procedures for all national registries worldwide. 14 national joint replacement registries were identified for inclusion in the study. 10 registries have nationwide coverage, with countries that mandate registry submission unsurprisingly showing the highest procedure completeness. Those that do not have full coverage strive towards it [12], and countries with lower procedure completeness and voluntary submission have plans to make submission of procedure to the register mandatory [12,18]. This well-trodden path by the more established registries is reflected in the general trend of improving procedure completeness over time. To our knowledge this paper is the first to collate procedure completeness data on national joint registries worldwide.

Overall procedure completeness for primary procedures across all years was high, with a median value of 96.5%. There were no real differences in procedure completeness between hip and knee replacements for registries that reported their completeness separately for either primary or revision procedures. Lower completeness was seen for the Canadian and Italian registries, which do not have full coverage or mandatory submission on a national level. Whilst mandating registry submission might be a useful tool to improve coverage, it should be emphasized that it is not a prerequisite for high completeness, as demonstrated by the AOANJRR and the SAR, which are entirely voluntary yet have full coverage and amongst the highest procedure completeness.

Procedure completeness data for revision procedures was slightly lower and more varied, with a median of 88.5%. This is to some extent expected as revision surgery has the potential to be necessary in the emergency setting, for example due to fracture, dislocation, or acute infection, or to be performed in different settings where data capture is more challenging. Unlike primary surgery, for it to be classed as valid it is also reliant on successful data linkage (including operative side) from previous procedures. Revisions without linkage risk being excluded from analysis, which would bias towards an underestimation of revision rates. This presents a challenge in the context of revision rate typically being the primary outcome measure for registries. However, we do not believe there is a minimal completeness threshold required to ensure results are useful. Ultimately completeness is an issue of missing data, where understanding how and why the data is missing, and the pattern of missingness, is the key determinant of its usefulness. For example, if missing data clusters within a surgeon, hospital, specific brand, or setting (e.g., indications such as trauma) this should be transparently reported, for example by stratifying completeness estimates by these characteristics.

### Completeness calculation methodology

The completeness estimate can be improved by using patient information to match individual procedures between datasets, a practice currently performed by 6 registries (Table 3). The denominator for the calculation is the union of the 2 datasets, and therefore, as in reality, completeness cannot exceed 100%. This is more resource-intensive but increases the validity of the estimate. Furthermore, matching procedures allows the possibility of further analysis such as capture–recapture models, which are used in epidemiology for estimating the size of a target population based on several incomplete lists of individuals; solely merging lists still misses those who are in the population but were missed by both lists. Capture–recapture models consider the duplicate, or matched, information between lists to estimate the number in the total population that were missed [13].

We used data available from the NAR’s annual report to conduct a capture–recapture analysis and compare it with the given completeness estimates. Interestingly the completeness of the comparison dataset, the Norwegian Patient Register (NPR), was lower than that of the registry (94.5% vs 97%), illustrating the limitations of assuming the hospital database alone as being complete and correct, as demonstrated in the NZJR, which has multiple years where completeness is more than 100% (28). Capture–recapture adjusted completeness estimates showed a minimal, 0.2% reduction for primary surgeries, and a 3.3% reduction in the completeness estimate for revision procedures in the NAR. The similarity of these estimates is reassuring for the validity of the completeness calculation for registries with similarly high completeness, as it is unlikely these differences reach clinical significance. Registries that use matched data for completeness calculations could include capture–recapture estimates as a sensitivity analysis to adjust their estimates.

Matching individual records between datasets rather than simply comparing procedural counts has the additional advantage of allowing data validation. Taking the AOANJRR as an example, procedural matching and pooling between the registry and a separately collected government dataset initially identifies procedures not registered in the other. Missing data is then populated, and data discrepancies settled through direct communication between the registry and relevant hospitals. Iterative processes such as these not only improve completeness but will be vital to ensuring data accuracy and therefore ultimately its quality.

### Terminology

Use of terminology was variable; completeness was expressed as coverage [16,17,23,27], compliance [21], and capture-rate [15], with one registry reporting “completeness” with reference to patient-reported outcome measures (PROMs) data collection [25]. There is clearly a need to standardize reporting terms across registries using easy to understand language that explains what is being estimated. We suggest completeness is used to describe the proportion of procedures captured by the registry compared with the true number of procedures performed in the population that year. As the term completeness has been used by some registries in relation to the availability of individual data points within each submitted record, for clarity we suggest that it is predicated by the concept being assessed, e.g., “procedure completeness,” “PROMs completeness.”

Coverage is useful when accurately described as it can indicate the possibility of geographical bias, or bias if procedures from the independent sector are not included for example. We suggest annual reports should include a brief description of coverage, i.e., the proportion of centers in the country that submitted data to the registry that year, compared with all centers in the country that performed a procedure in that year.

These suggestions to standardize terminology would benefit from further discussion between registry representatives at forums such as ISAR in order to form international consensus.

### Limitations

The main limitation of this study is that coverage and completeness are not the only quality indicators for national registry data; a replete registry might of course still contain inaccurate data. As discussed, this will require registries to have mechanisms in place for individual procedural matching, analysis, and resolution of discrepancies. The process of data quality assurance would also benefit from standardization and international consensus. Once established, maintenance of standards could be maintained by accreditation through an independent organization such as ISAR. This would need to be supported by significant investment in resources but is arguably increasingly necessary as the reliance on registry data grows. Finally, there was no study protocol prior to the initiation of this study.

### Conclusion

National hip and knee replacement registry data generally reflects excellent coverage (full in 10 of 14 registries) and completeness (primary procedures 96.5% and revisions 88.5%) over the last 2 decades. Results varied largely depending on coverage and whether registry submission was mandatory, but with a general trend of individual improvement over time as registries become more established. The validity of completeness calculation methodology varied between registries.

*In perspective,* completeness calculation methodology can be improved by using matched data and capture–recapture analysis to estimate the number of procedures missed by both datasets. Standardized definitions of terms such as coverage and completeness have been provided, with international collaboration, such as through ISAR, recommended to facilitate consistent quality reporting. Overall, these results support registries as valuable tools for conducting post-market surveillance of medical devices and providing quality assurance.

## Notes

JMRF: data collection, methodology, analysis, visualization, writing—original draft. KD: investigation, resources, writing—review and editing, supervision. MRW: methodology, resources, writing—review and editing, supervision. DJP: visualization, writing—review and editing, supervision. EC, ERB, JW, MT, RV: data collection, investigation, writing—review and editing. AL, AMF, GH, KM, OF, SO: data validation, investigation, writing—review and editing. OR: investigation, writing—original draft, writing—review and editing, supervision. AS: conceptualization, data validation, methodology, analysis, visualization, writing—review and editing, supervision.

The authors would like to thank the registries included in this study for their time, cooperation, and transparency in reporting data, and Kate Powell for assisting with data validation. They would also like to extend their sincere thanks to Prof Stephen Graves, Dr Claus Varnum, and Dr Bart Pils for peer-reviewing this work and substantially improving it through their comments.

Handling co-editor: Bart A. Swierstra

*Acta* thanks Stephen Ellis Graves, Bart G Pijls, and Claus Varnum for help with peer review of this manuscript.

## APPENDIX

### Capture recapture calculation from the NAR report 2023, 2019–2020 data for hip replacements

NAR = Norwegian Arthroplasty Register

NPR = Norwegian Patient Register

NAR compliance (%) =

 cases only in NAR (x) + cases in both NAR and NPR (z)

 cases only in NAR (x) + cases only in NPR (y) + cases in both (z)

NPR compliance (%) =

 cases only in NPR (y) + cases in both NAR and NPR (z)

 cases only in NAR (x) + cases only in NPR (y) + cases in both (z)

NAR compliance = 97%

NPR compliance = 94.5%

Total cases = 19,190

Therefore x + y + z = 19,190

0.97 = (x + z) / 19,190

z = 18,614 – x

0.945 = (y + z) / 19,190

z = 18,135 – y

{x + y + z = 19,190, z = 18,135 – y, z = 18,614 – x}

x = 1,055, y = 576, z = 17,559

Cases only in NAR (x) = 1,055

Cases only in NPR (y) = 576

Cases in both NAR and NPR (z) = 17,559

Capture recapture N = MT/R

N = total population

M = number in first source = x + z = 18,614

R = number in both sources = z = 17,559

T = number in second source = both + only NPR = y + z = 18,135

N = (18,614 × 18,135) /17,559

Total population estimate from capture-recapture (N) = 19,225

This estimate adds 35 cases to the population.

Adjusted completeness = 18,614 / 19,225 = 96.8%

Little difference from given figure of 97.0%.

**For revision operations**

x + y + z = 3,178

0.908 = (x + z) / 3178

z = 2,886 – x

0.735 = (y + z) / 3,178

z = 2,336 – y

{x + y + z = 3,178, z = 2,886 – x, z = 2,336 – y}

x = 842, y = 292, z = 2,044

M = 2,886, R = 2,044,T = 2,336

N = (2,886 × 2,336) / 2,044

Total population estimate from capture-recapture (N) = 3,298

This estimate adds 190 cases to the population.

Adjusted completeness = 2,886/3,298 = 87.5%

Small difference from given figure of 90.8%
